# Association of neutrophil to lymphocyte ratio and D-dimer with functional outcome in patients with cerebral venous sinus thrombosis

**DOI:** 10.1186/s12883-022-03030-4

**Published:** 2023-01-19

**Authors:** Rui Sun, Feihong Huang, Wen Wu, Ge Yin, Qichao Ding, Zhengsheng Gu, Cunxiu Fan, Chenrui Song, Meng Liang, Xiaobei Liu, Xiaoying Bi

**Affiliations:** 1 Department of Neurology, Shanghai Changhai Hospital, Second Military Medical University/Naval Medical University, Shanghai, 200433 China; 2 Department of Neurology, Guilin People’s Hospital, Guilin, 541000 China

**Keywords:** Neutrophil to lymphocyte ratio (NLR), D-dimer, Cerebral venous sinus thrombosis, Outcome

## Abstract

**Background:**

Investigations on the risk factors for the prognosis of cerebral venous sinus thrombosis (CVST) are limited. This study aimed to explore whether specific inflammatory factors and coagulation indictors are associated with functional outcome in patients treated for CVST.

**Methods:**

This retrospective study included 137 patients admitted to our hospital between January 2010 and October 2021. The functional outcome was assessed with the modified Rankin Scale (mRS) score at discharge. Patients were divided into two groups, 102 patients with favorable outcomes (mRS 0-1) and 35 patients with poor outcomes (mRS 2-6). The clinical indexes were compared between two groups. Multivariable logistic regression was performed to identify the independent influencing factors for poor outcomes of CVST patients. The prognostic indicators were analyzed using the receiver operating characteristic (ROC) curve.

**Results:**

Compared with the favorable outcome group, the incidence of impaired consciousness and brain lesion, the levels of D-dimer, RDW, neutrophil count, neutrophil to lymphocyte ratio (NLR) and red blood cell distribution width to platelet ratio (%) on admission were significantly higher in the poor outcome group, while the level of lymphocyte count was significantly lower. After multivariable logistic regression analysis, baseline D-dimer level (odds ratio (*OR*), 1.180; 95% confidence interval (*CI*), 1.019-1.366, *P* = 0.027) and NLR (*OR*, 1.903; 95%*CI*, 1.232-2.938, *P* = 0.004) were significantly associated with unfavorable outcome at discharge. The ROC curve analysis showed that the areas under the curve of D-dimer, NLR and their combined detection for predicting worse outcome were 0.719, 0.707 and 0.786, respectively.

**Conclusions:**

Elevated D-dimer level and NLR on admission were associated with an increased risk of poor functional outcome in patients with CVST.

**Supplementary Information:**

The online version contains supplementary material available at 10.1186/s12883-022-03030-4.

## Introduction

Cerebral venous sinus thrombosis (CVST) is an uncommon manifestation of thrombosis that occurs mainly in younger individuals aged 20 to 50 years [1, 2]. Although earlier diagnosis and advances made in the therapeutic management of CVST have improved its prognosis, many patients still suffer from chronic residual symptoms such as headache, motor deficits, impaired vision or cognitive impairment, while some patients retain severe dependency or die [3, 4]. A multicenter study with 1144 patients indicated that 78.4% presented with complete recovery (defined as the modified Rankin Scale (mRS) 0 to 1) and 10.0% presented with death or dependency (defined as mRS 3 to 6) [5]. Therefore, it is essential to explore convenient and reliable predictive indicators for functional outcomes and provide potential therapeutic targets to improve the prognosis.

Previous studies on thrombotic diseases support the role of inflammation in the pathophysiology of thrombosis, particularly in initiation and amplification of coagulation [6]. In experimental models of cerebral venous thrombosis, inflammatory cellular infiltration has been noted at the sites of thrombosis [7]. Monocytes and neutrophils were reported to provide the initiating stimulus for the development of venous thrombosis [7, 8]. Recently, inflammatory biomarkers like neutrophil to lymphocyte ratio (NLR), lymphocyte to monocyte ratio (LMR), platelet to lymphocyte ratio (PLR) and red blood cell distribution width to platelet ratio (RPR) have been confirmed to be related to the outcomes of various inflammatory or vascular diseases, such as stroke, sepsis and myocardial infarction [9–13]. Nonetheless, research focused on the role of these inflammatory factors in predicting the prognosis of CVST remains limited.

As a soluble fibrin degradation product caused by the breakdown of thrombus, D-dimer has been reported to work as a diagnostic factor in CVST [14, 15]. Recent studies have shown that D-dimer levels are independently associated with the risk of intracranial hemorrhage after CVST [16]. However, whether D-dimer could be a valuable prognostic indicator for CVST remains unclear.

The present study aimed to identify the association between inflammatory indicators and D-dimer with short-term functional outcome in patients with CVST to estimate their predictive value as prognostic factors.

## Methods

### Study design

**Fig. 1:**
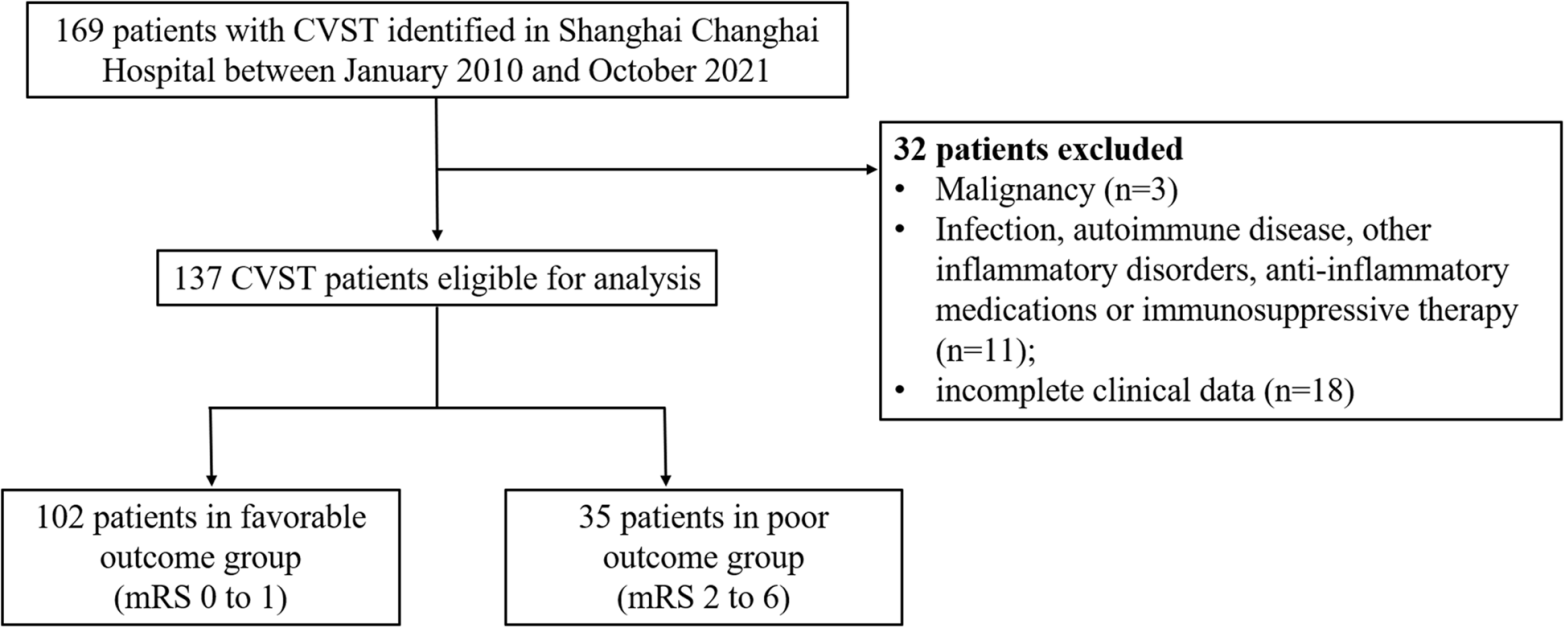
Flow chart of the study

We retrospectively included patients with the diagnosis of CVST admitted to our hospital between January 2010 and October 2021. The inclusion criteria were diagnosed CVST patients based on magnetic resonance imaging (MRI) and magnetic resonance venography (MRV), or computed tomographic venography (CTV), or digital subtraction angiography (DSA) [17]. Patients were excluded if they were diagnosed with the following conditions: hepatic or renal failure, malignancy, infection at admission, autoimmune disease, other inflammatory disorders, anti-inflammatory medications or immunosuppressive therapy (Fig. 1). This study was approved by the Ethics Committee of Changhai Hospital, Shanghai, China.

### Data collection

Medical records of all patients were reviewed, including demographic data and clinical characteristics, radiological evaluations, laboratory indicators, and functional outcomes. Type of onset was defined as acute (duration of symptoms less than 2 days on admission), subacute (duration of symptoms between 2 days and 30 days), and chronic (duration of symptoms longer than 30 days) [5]. The number of involved sinuses, localizations of thrombus and lesions in CT or MRI were identified by two experienced radiologists. The blood samples were routinely obtained on admission and NLR, LMR, PLR, and RPR were calculated. The calculation formulas of these indexes were as follows: NLR = neutrophil count/lymphocyte count; LMR = lymphocyte count/monocyte count; PLR, platelet count/lymphocyte count; RPR (%) = red blood cell distribution width/platelet count × 100%. Clinical outcome was evaluated by the mRS at discharge: mRS 0-1 classified as favorable outcome, mRS 2-6 classified as poor outcome [18, 19].

### Statistical analysis

Quantitative data were described as mean ± standard deviation or median with interquartile range (IQR), and analyzed by independent Student’s *t* test or Mann-Whitney *U* test. Categorical data were described as frequency and percentage (%), and analyzed by *χ2* test or Fisher’s exact test. Variables with *P* value < 0.05 in the comparison between two groups were subjected to collinearity test, and variables with variance inflation factor (VIF) < 10 and tolerance > 0.1 were extracted by multivariable logistic regression analysis (Supplemental Table 1). Multivariable logistic regression analysis was applied to identify possible risk factors associated with the outcome of CVST. The correlations between the mRS score and significant variables were analyzed by the Pearson correlation test. The receiver operating characteristic (ROC) curve was used to assess the sensitivity and specificity of significant indicators and the optimal cut-off values for predicting the outcome. *P* value < 0.05 was considered statistically significant. All statistical analysis were performed with SPSS statistical package (Version 22.0, Armonk, NY, USA).

## Results

### Baseline Characteristics of the two cohorts

A total of 137 patients with CVST were included in the analysis. The demographic and clinical characteristics of the patients are shown in Table 1. The patients were divided into two groups: a ‘favorable outcome’ group (n = 102, mean age: 38.27 ± 15.07 years, female: 45.1%) and a ‘poor outcome’ group (n = 35, mean age: 43.57 ± 14.98 years, female: 54.3%). In the favorable outcome group, 4.9% of patients were in the acute stage, 65.7% in the subacute stage, and 29.4% in the chronic stage; in the poor outcome group, 17.1% of patients were in the acute stage, 60.0% in the subacute stage, and 22.9% in the chronic stage.

**Table 1:** Baseline characteristics of the two cohorts

Variable		Favorable outcome		Poor outcome		***t/χ***^***2***^***/Z***		***P*** value
		*n*=102		*n*=35				
**Age (years; mean ± SD)**		38.27 ± 15.07		43.57 ± 14.98		-1.797		0.075
**Gender (n,%)**						0.882		0.433
Male		56 (54.9%)		16 (45.7%)				
Female		46 (45.1%)		19 (54.3%)				
**Onset (n,%)**						5.398		0.067
Acute		5 (4.9%)		6 (17.1%)				
Subacute		67 (65.7%)		21 (60.0%)				
Chronic		30 (29.4%)		8 (22.9%)				
**Etiology and risk factors (n,%)**								
Oral contraceptives		10 (9.8%)		0 (0.0%)		2.394		0.122
Pregnancy, abortion or puerperium		12 (11.8%)		7 (20.0%)		0.870		0.351
Thrombophilia		12 (11.8%)		3 (8.6%)		0.043		0.835
Hematological diseases		7 (6.9%)		2 (5.7%)		<0.001		1.000
Physical factors		2 (2.0%)		1 (2.9%)		<0.001		1.000
**Clinical manifestation (n,%)**								
Headache		85 (83.3%)		24 (68.6%)		3.492		0.062
Visual disturbance		16 (15.7%)		4 (11.4%)		0.379		0.596
Seizure		20 (19.6%)		12 (34.3%)		3.136		0.104
Sensory disorder		3 (2.9%)		2 (5.7%)		0.570		0.602
Motor deficit		14 (13.7%)		8 (22.9%)		1.612		0.204
Aphasia		3 (2.9%)		1 (2.9%)		—		1.000^a^
Mental disturbance		3 (2.9%)		2 (5.7%)		—		0.602^a^
Impaired consciousness		5 (4.9%)		8 (22.9%)		7.803		0.005*
**Brain lesion (n,%)**								<0.001*^a^
Cerebral hemorrhage		29 (28.4%)		12 (34.3%)				
Subarachnoid hemorrhage		12 (11.8%)		2 (5.7%)				
Cerebral infarction		26 (25.4%)		12 (34.3%)				
Hemorrhagic infarction		2 (2.0%)		7 (20.0%)				
No lesion		33 (32.4%)		2 (5.7%)				
**Number of sinuses involved (n,%)**						1.996		0.591
1 sinus		32 (31.4%)		10 (28.6%)				
2 sinuses		33 (32.4%)		12 (34.3%)				
3 sinuses		25 (24.5%)		6 (17.1%)				
More than 3 sinuses		12 (11.8%)		7 (20.0%)				
**Localization of thrombus (n,%)**								
Superior sagittal sinus		47 (46.1%)		22 (62.9%)		2.935		0.117
Inferior sagittal sinus		7 (6.9%)		5 (14.3%)		0.988		0.320
Transverse sinus		74 (72.5%)		23 (65.9%)		0.589		0.519
Sigmoid sinus		61 (59.8%)		19 (54.3%)		0.327		0.691
Straight sinus		13 (12.7%)		9 (25.7%)		3.252		0.107
Jugular vein		2 (2.0%)		1 (2.9%)		—		1.000^a^
Cortical veins		1 (1.0%)		1 (2.9%)		—		0.447^a^

* *P* < 0.05; ^a^ Fisher's exact probability test

Compared with the favorable outcome group, impaired consciousness was significantly more common among patients in the poor outcome group (22.9% vs. 4.9%, *P* = 0.005). Brain parenchymal lesion was also significantly more frequent in the poor outcome group (94.3% vs. 67.6%, *P* < 0.001). There were no significant differences in other clinical symptoms (headache, visual disturbance, seizure, focal deficits and mental disturbance), etiology and risk factors, number of sinuses involved, and localization of thrombus.

### Laboratory indicators of the two groups

Compared to patients with favorable outcomes, D-dimer level (2.16 [1.27-8.88] vs. 0.78 [0.40-1.93], *P* < 0.001), neutrophil count (6.04 [4.70-9.67] vs. 4.90 [3.42-7.32], *P* = 0.011), red cell distribution (RDW) (%) (13.40 [12.70-14.70] vs. 12.80 [12.08-13.60], *P* = 0.012), NLR (4.09 [2.45-6.92] vs. 2.76 [1.97-3.64], *P* < 0.001) and RPR (%) (7.16 [5.97-10.00] vs. 6.15 [4.45-7.28], *P* = 0.001) was significantly higher in patients with poor outcomes, while lymphocyte count was significantly lower in patients with poor outcomes (1.41 [0.93-2.10] vs. 1.92 [1.44-2.19], *P* = 0.011). No significant differences were observed in the other laboratory indicators obtained (Table 2).

**Table 2:** Laboratory indicators of the two groups

Variable		Favorable outcome		Poor outcome		***t/Z***		***P*** value
		*n*=102		*n*=35				
D-dimer (mg/L; M, IQR)		0.78 (0.40-1.93)		2.16 (1.27-8.88)		-3.865		<0.001*
PT (s; M, IQR)		13.60 (13.08-14.43)		13.80 (13.00-14.90)		-0.499		0.618
APTT (s; M, IQR)		37.15 (34.60-39.43)		36.30 (31.70-41.50)		-0.624		0.532
TT (s; M, IQR)		16.25 (15.48-17.50)		16.50 (15.20-18.30)		-0.632		0.527
Fibrinogen (g/L; M, IQR)		3.31 (2.89-4.14)		3.07 (2.81-3.98)		-0.859		0.390
TC (mmol/L; mean ± SD)		4.54 ± 1.26		4.93 ± 1.33		-1.532		0.128
TG (mmol/L; M, IQR)		1.36 (1.05-1.82)		1.36 (0.94-1.83)		-0.38		0.704
LDL-C (mmol/L; M, IQR)		2.69 (2.14-3.08)		2.94 (2.19-3.75)		-1.291		0.197
HDL-C (mmol/L; mean ± SD)		1.16 (1.01-1.38)		1.27 (1.16-1.63)		-1.893		0.058
Neutrophil (× 10^9^/L; M, IQR)		4.90 (3.42-7.32)		6.04 (4.70-9.67)		-2.539		0.011*
Lymphocyte (× 10^9^/L; M, IQR)		1.92 (1.44-2.19)		1.41 (0.93-2.10)		-2.537		0.011*
Monocyte (× 10^9^/L; M, IQR)		0.59 (0.44-0.78)		0.50 (0.38-0.81)		-0.849		0.396
PLT (× 10^9^/L; M, IQR)		225.00 (182.75-289.25)		202.00 (141.00-280.00)		-1.668		0.095
Hb (g/L; M, IQR)		138.00 (124.00-154.25)		132.00 (111.00-154.00)		-1.007		0.314
MCV (fL; M, IQR)		88.90 (85.48-91.20)		88.70 (84.30-92.10)		-0.284		0.777
MPV (fL; M, IQR)		10.20 (9.50-10.90)		10.50 (9.40-11.00)		-0.61		0.542
MCHC (g/L; mean ± SD)		336.08 ± 16.23		333.74 ± 16.45		0.732		0.465
RDW (%; M, IQR)		12.80 (12.08-13.60)		13.40 (12.70-14.70)		-2.526		0.012*
NLR (M, IQR)		2.76 (1.97-3.64)		4.09 (2.45-6.92)		-3.65		<0.001*
LMR (M, IQR)		3.26 (2.43-4.34)		3.00 (1.66-4.63)		-0.548		0.584
PLR (M, IQR)		125.32 (91.92-162.13)		152.38 (74.89-261.54)		-0.819		0.413
RPR (%; M, IQR)		6.15 (4.45-7.28)		7.16 (5.97-10.00)		-3.361		0.001*
* *P* < 0.05								

*PT*, prothrombin time, *APTT*, activated partial thromboplastin time, *TT*, thrombin time, *TC*, total cholesterol, *TG*, triglyceride, *LDL-C*, low density lipoprotein cholesterol, *HDL-C*, high density lipoprotein cholesterol, *Hb*, hemoglobin, *MCV*, mean corpuscular volume, *MPV*, mean platelet volume, *MCHC*, mean corpuscular hemoglobin concentration, *RDW*, red cell distribution width, *PLT*, platelet, *NLR*, neutrophil to lymphocyte ratio, *LMR*, lymphocyte to monocyte ratio, *PLR*, platelet to lymphocyte ratio, *RPR*, red blood cell distribution width to platelet ratio

### The correlation between clinical indicators and short-term outcome

The correlation between the functional outcome evaluated by mRS score and significant clinical indicators is described in Table 3. The results of the analysis showed that impaired consciousness (*rho* = 0.267, *P* = 0.002), D-dimer level (*rho* = 0.388, *P* < 0.001), neutrophil count (*rho* = 0.219, *P* = 0.010), NLR (*rho* = 0.419, *P* < 0.001) and RPR (%) (*rho* = 0.308, *P* < 0.001) were positively associated with mRS score at discharge, among which NLR had the closest correlation.

**Table 3:** The correlation between clinical indicators and the functional outcome

Variable	***rho*** value	***P*** value
Impaired consciousness	0.267	0.002*
Brain lesion	-0.116	0.178
D-dimer	0.388	<0.001*
RDW	-0.021	0.806
Neutrophil	0.219	0.010*
Lymphocyte	-0.142	0.099
NLR	0.419	<0.001*
RPR (%)	0.308	<0.001*

*RDW*, red cell distribution width, *NLR*, neutrophil to lymphocyte ratio, *RPR*, red blood cell distribution width to platelet ratio

* *P* < 0.05

### Independent risk factors for poor outcome in patients with CVST

Potentially significant variables in Table 1 and Table 2 were extracted as follows: impaired consciousness (*P* = 0.005), brain lesion (*P* < 0.001), D-dimer (*P* < 0.001), RDW (*P* = 0.012), neutrophil count (*P* = 0.011), lymphocyte count (*P* = 0.011), NLR (*P* < 0.001), RPR (%) (*P* = 0.001). These potentially significant variables were assessed for collinearity however none was found for these variables (Supplementary Table 1). Following, we conducted multivariable logistic regression analysis to assess the associations between these factors and the functional outcome at discharge. The results demonstrated that D-dimer level (odds ratio (*OR*), 1.180; 95% confidence interval (*CI*), 1.019-1.366, *P* = 0.027) and NLR (*OR*, 1.903; 95%*CI*, 1.232-2.938, *P* = 0.004) were independent risk factors for poor functional outcome in CVST patients (Table 4).

**Table 4:** Multivariable logistic regression analysis for poor outcomes of CVST patients

A.Variable	***OR***	95%***CI***	***P*** value
Impaired consciousness	0.302	0.068-1.333	0.114
Brain lesion	0.835	0.600-1.163	0.286
D-dimer	1.180	1.019-1.366	0.027*
RDW	0.978	0.927-1.032	0.422
Neutrophil	0.805	0.613-1.057	0.118
Lymphocyte	1.885	0.816-4.352	0.138
NLR	1.903	1.232-2.938	0.004*
RPR (%)	1.222	0.996-1.500	0.054

*RDW*, red cell distribution width, *NLR*, neutrophil to lymphocyte ratio, *RPR*, red blood cell distribution width to platelet ratio, *OR*, odds ratio, *CI*, confidence interval

* *P* < 0.05

### ROC curve analysis of single and combined detection of baseline D-dimer and NLR

**Fig. 2:**
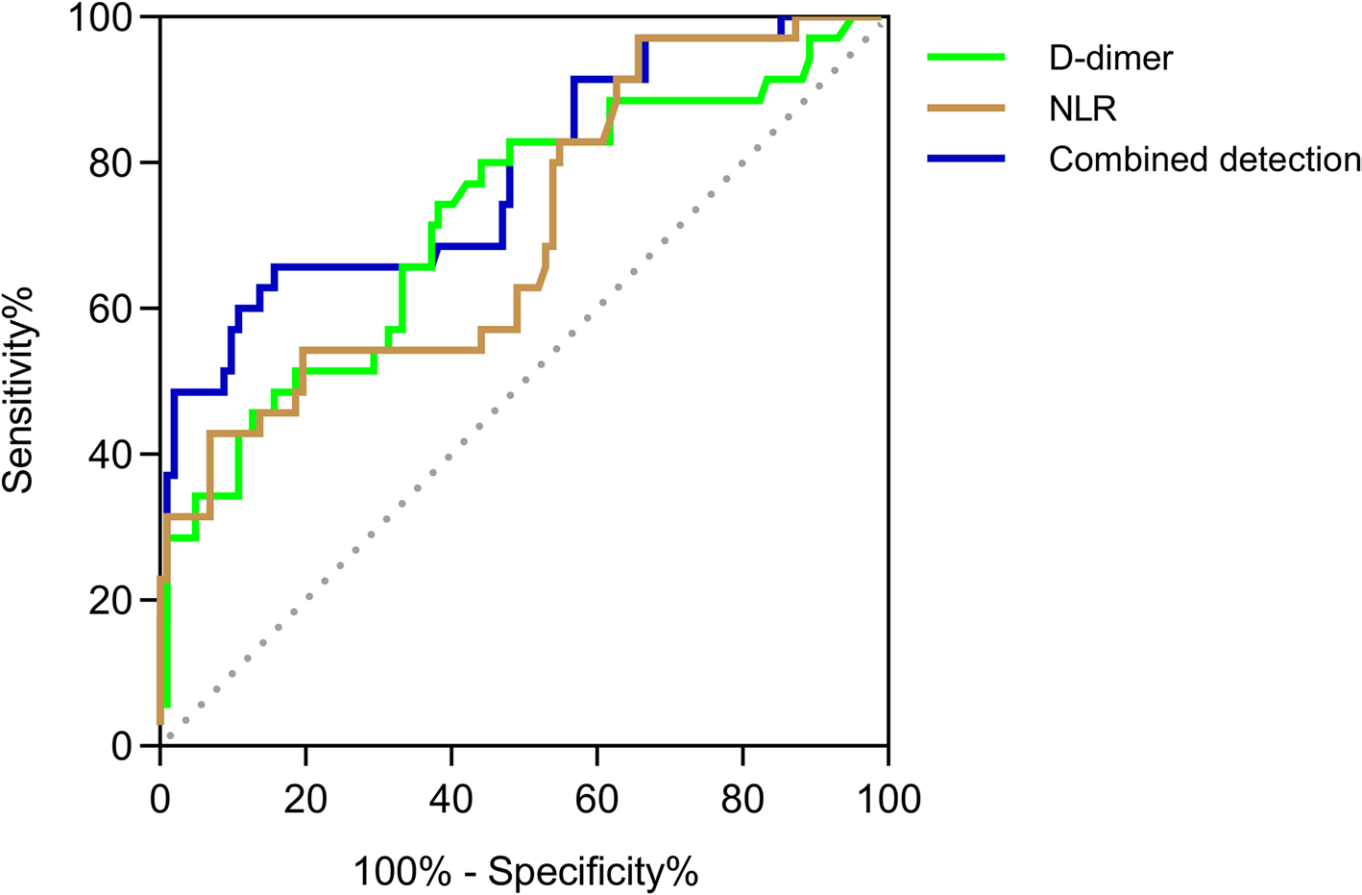
Receiver operating characteristic curve of single and combined detection of D-dimer and NLR on predicting the functional outcome of CVST. NLR, neutrophil to lymphocyte ratio; CVST, cerebral venous sinus thrombosis

The ROC curve showed that D-dimer level and NLR on admission could statistically predict the short-term outcome of CVST (Fig. 2). The area under the curve (AUC) of D-dimer was 0.719 and the optimal cut-off value was 1.335 (sensitivity 74.3%, specificity 61.8%, YI 0.361). The AUC of NLR was 0.707 and the optimal cut-off value was 5.170 (sensitivity 42.9%, specificity 93.1%, YI 0.360). In addition, the combined detection of two indexes showed higher predictive power, with an AUC of was 0.786. The optimal cut-off value was 9.650 (sensitivity 65.7%, specificity 84.3%, YI 0.500) (Table 5).

**Table 5:** ROC curve analysis of single and combined detection of D-dimer and NLR

Index	AUC	Cut-off value	95% ***CI***	Sensitivity(%)	Specificity(%)	YI
D-dimer	0.719	1.335	0.616~0.823	74.3	61.8	0.361
NLR	0.707	5.170	0.605~0.809	42.9	93.1	0.360
Combination	0.786	9.650	0.693~0.879	65.7	84.3	0.500

*NLR*, neutrophil to lymphocyte ratio, *AUC*, area under curve, *CI*, confidence interval, *YI*, Youden’s index

## Discussion

The results of our study indicated that baseline NLR and D-dimer level may be independently associated with the functional outcome at the point of discharge for patients with CVST. We also observed that the combined application of these two indexes had high predictive value for the neurological recovery after CVST.

Although the mortality rate associated with CVST has dramatically decreased in recent years, local brain lesions followed by herniation or seizures can be fatal [1]. Intracranial hemorrhage on neuroimaging was reported to be a marker for worse prognosis of CVST [2]. Moreover, it was observed that in patients with CVST, altered consciousness at admission was an independent predictor of poor functional outcome [20, 21]. Both intracranial hemorrhage and GCS score < 9 on admission were associated with death and dependency [22]. In the present study, it was also observed that increased local brain lesions and impaired consciousness were associated with unfavorable outcome, consistent with previous findings [22–24].

Data from preclinical and clinical studies on CVST have suggested that the increase in venous and capillary pressure after local venous occlusion could lead to the disruption of the blood brain barrier and vasogenic edema [25]. The increased intravenous pressure may finally result in decreased cerebral blood flow, failure of cellular metabolism, and cytotoxic edema [25]. The reduction of oxygen and failure of energy metabolism consequently triggers a local inflammatory immune response [26, 27]. Inflammation has been reported to closely correlate with venous thrombosis, which participates in the amplification of coagulation and shifts the hemostatic balance towards a prothrombotic state [6, 28]. During the inflammatory process, the release of plasminogen activators is increased with subsequent plasmin generation [6], which may suggest the relationship between extension of thrombosis and increased inflammatory markers. In the acute phase, neutrophil counts elevate and lymphocyte counts decrease, which could be the primary nonspecific reaction of the immune system [29]. It was also reported that after the initiating stimuli of neutrophil, platelets aggregate in the core of the thrombus, produce proinflammatory molecules, and lead to the formation and progression of pathological thrombosis [30, 31]. As a measurement of the size variation of circulating red blood cells, RDW was also associated with vein thrombosis, ischemic stroke and intracranial hemorrhage after CVST [16, 32]. Consistent with these findings, our results also demonstrated significantly higher levels of neutrophil count and RDW, and lower levels of lymphocyte counts in patients with poor outcome.

The NLR, PLR, LMR and RPR are calculated ratios from the lymphocyte, neutrophil, platelet counts and RDW, which are conveniently obtained from standard blood samples. Previous studies have indicated associations between NLR, LMR and PLR with CVST, suggesting these inflammatory factors may have predictive value for the presence of CVST [30, 33, 34]. In addition, RPR, a novel potential indicator of inflammatory processes, has been shown to independently predict poor prognosis after spontaneous intracerebral hemorrhage [35]. However, studies investigating the role of these indicators in relation to the clinical outcome of CVST patients remain limited. Recently, Wang et al. reported that higher NLR in CVST patients was significantly related to poor short-term outcome [19]. Our findings also demonstrated a significant positive correlation between NLR, RPR and the short-term outcome after CVST, suggesting a more common inflammatory state in patients with poor outcome. To our knowledge, this is the first study to propose that elevated RPR may correlate with unfavorable prognosis in CVST patients. Since the multivariable logistic regression analysis found no statistical significance, further investigations on the potential association of RPR with the outcome of CVST are required. In addition, we verified that elevated NLR was an independent prognostic indicator. The ROC curve showed that baseline NLR was statistically predictive of the functional outcome of CVST, with an AUC of 0.707 and an optimal cut-off value of 5.170.

D-dimer is a specific degradation product of the cross-linked fibrin after being degraded by plasmin, reflecting the activation of the coagulation system and fibrinolysis [31, 36]. Several studies have observed that D-dimer elevation was associated with diverse thrombotic vascular conditions and inflammatory conditions [37]. D-dimer has been widely used in the diagnosis of CVST [38]. Moreover, high D-dimer levels correlate with greater thrombus extension and risk of intracranial hemorrhage in patients with CVT [15, 16]. Our study further confirms that elevated D-dimer level on admission is independently associated with poor recovery. In addition, D-dimer was found to have an AUC of 0.719, with a sensitivity of 74.3% and a specificity of 61.8%, which may be a valuable marker for predicting the short-term outcome.

Although D-dimer was reported to be sensitive in CVST, its specificity was moderate, and therefore could return false positive results in various conditions such as pregnancy or the postpartum state, malignancy or as result of other inflammatory process [39]. Furthermore, our result showed relatively low sensitivity for NLR compared to its high specificity. To further improve its predictive value, we combined these two indicators and found that the combined detection of NLR and D-dimer had an AUC of 0.786, a sensitivity of 65.7%, a specificity of 84.3%, and its efficacy was superior to that of a single detection. Therefore, we propose that NLR and D-dimer could be detected together to improve the accuracy of predicting poor prognosis in patients with CVST. In addition, these results suggest that anticoagulant therapy accompanied by anti-inflammatory drugs at an early stage in treatment may further improve the functional outcome of CVST, however further studies are needed to confirm this.

Our study has several limitations. Firstly, due to the retrospective and single-center nature of this study, the sample size was relatively small and some patients were missing data. Secondly, although relative strict inclusion and exclusion criteria were adopted, heterogeneity in the sample remained which may bias the results. Thirdly, the association between these indicators and the long-term functional outcome in patients treated for CVST needs to be further explored in longitudinal research.

## Conclusions

In CVST patients, high D-dimer level and NLR on admission were found to be associated with an increased risk of poor functional outcome at discharge. In addition, these two indicators might have predictive value for the short-term prognosis of CVST. Further research on the mechanisms underpinning these observations are required in order to identify the appropriate therapeutic strategies for these patients.

## Supplementary Information

**Additional file 1:** **Supplementary Table 1.** Collinearity test for clinical indexes

## Data Availability

The datasets used during the current study are available from the corresponding author on reasonable request.

## Abbreviations

APTT: Activated partial thromboplastin time
Apo: Apolipoprotein
AUC: Area under the curve
CI: Confidence interval
CVST: Cerebral venous sinus thrombosis
Hb: Hemoglobin
HDL-C: High density lipoprotein cholesterol
LDL-C: Low density lipoprotein cholesterol
LMR: Lymphocyte to monocyte ratio
MCHC: Mean corpuscular hemoglobin concentration
MCV: Mean corpuscular volume
MPV: Mean platelet volume
mRS: The modified Rankin Scale
MRV: Magnetic resonance venogram
NLR: Neutrophil to lymphocyte ratio
OR: Odds ratio
PLR: Platelet to lymphocyte ratio
PLT: Platelet
PT: Prothrombin time
RDW: Red cell distribution width
ROC: Receiver operating characteristic
RPR: Red blood cell distribution width to platelet ratio
TC: Total cholesterol
TG: Triglyceride
TT: Thrombin time.

## References

[1] Capecchi M, Abbattista M, Martinelli I (2018). Cerebral venous sinus thrombosis. J Thromb Haemost..

[2] Silvis SM, de Sousa DA, Ferro JM, Coutinho JM (2017). Cerebral venous thrombosis. Nat Rev Neurol..

[3] Devasagayam S, Wyatt B, Leyden J, Kleinig T (2016). Cerebral Venous Sinus Thrombosis Incidence Is Higher Than Previously Thought: A Retrospective Population-Based Study. Stroke.

[4] Hiltunen S, Putaala J, Haapaniemi E, Tatlisumak T (2016). Long-term outcome after cerebral venous thrombosis: analysis of functional and vocational outcome, residual symptoms, and adverse events in 161 patients. Journal of Neurology.

[5] Duman T, Uluduz D, Midi I, Bektas H, Kablan Y, Goksel BK (2017). A Multicenter Study of 1144 Patients with Cerebral Venous Thrombosis: The VENOST Study. J Stroke Cerebrovasc Dis..

[6] Donadini MP, Riva N, Ageno W (2015). Epidemiology and pathophysiology of venous thromboembolism: similarities with atherothrombosis and the role of inflammation. Thrombosis and Haemostasis.

[7] von Bruhl ML, Stark K, Steinhart A, Chandraratne S, Konrad I, Lorenz M (2012). Monocytes, neutrophils, and platelets cooperate to initiate and propagate venous thrombosis in mice in vivo. J Exp Med..

[8] Heestermans M, Salloum-Asfar S, Salvatori D, Laghmani el H, Luken BM, Zeerleder SS (2016). Role of platelets, neutrophils, and factor XII in spontaneous venous thrombosis in mice. Blood..

[9] Cicek G, Acikgoz SK, Bozbay M, Altay S, Ugur M, Uluganyan M (2015). Neutrophil-lymphocyte ratio and platelet-lymphocyte ratio combination can predict prognosis in patients with ST-segment elevation myocardial infarction undergoing primary percutaneous coronary intervention. Angiology.

[10] Wang F, Hu S, Ding Y, Ju X, Wang L, Lu Q (2016). Neutrophil-to-Lymphocyte Ratio and 30-Day Mortality in Patients with Acute Intracerebral Hemorrhage. J Stroke Cerebrovasc Dis..

[11] Ge S, Lin S, Zhang L, Zeng M (2020). The Association of Red Blood Cell Distribution Width to Platelet Count Ratio and 28-Day Mortality of Patients with Sepsis: A Retrospective Cohort Study. Ther Clin Risk Manag..

[12] Ren H, Liu X, Wang L, Gao Y (2017). Lymphocyte-to-Monocyte Ratio: A Novel Predictor of the Prognosis of Acute Ischemic Stroke. J Stroke Cerebrovasc Dis..

[13] Park MG, Kim MK, Chae SH, Kim HK, Han J, Park KP (2018). Lymphocyte-to-monocyte ratio on day 7 is associated with outcomes in acute ischemic stroke. Neurol Sci..

[14] Meng R, Wang X, Hussain M, Dornbos D, Meng L, Liu Y (2014). Evaluation of plasma D-dimer plus fibrinogen in predicting acute CVST. Int J Stroke.

[15] Hiltunen S, Putaala J, Haapaniemi E, Salonen O, Tatlisumak T (2013). D-dimer and clinicoradiologic features in cerebral venous thrombosis. J Neurol Sci..

[16] Ling J, Fang M, Wu Y (2022). Association of red cell distribution width and D-dimer levels with intracranial hemorrhage in patients with cerebral venous thrombosis. Clin Neurol Neurosurg..

[17] Ferro JM, Aguiar de Sousa D (2019). Cerebral Venous Thrombosis: an Update. Curr Neurol Neurosci Rep..

[18] Liao CH, Liao NC, Chen WH, Chen HC, Shen CC, Yang SF (2020). Endovascular Mechanical Thrombectomy and On-Site Chemical Thrombolysis for Severe Cerebral Venous Sinus Thrombosis. Sci Rep..

[19] Wang L, Duan J, Bian T, Meng R, Wu L, Zhang Z (2018). Inflammation is correlated with severity and outcome of cerebral venous thrombosis. J Neuroinflammation.

[20] Gupta S, Soni R, Dhull P, Somasekharan M, Sreen A (2021). Glasgow Coma Scale ≤ 12 at Admission is a Predictor of Poor Functional Outcome (mRS 2–6) at One Year in Patients with Cerebral Venous Thrombosis. Journal of Stroke and Cerebrovascular Diseases.

[21] Sassi SB, Touati N, Baccouche H, Drissi C, Romdhane NB, Hentati F (2017). Cerebral Venous Thrombosis: A Tunisian Monocenter Study on 160 Patients. Clin Appl Thromb Hemost..

[22] Pongmoragot J, Saposnik G (2012). Intracerebral hemorrhage from cerebral venous thrombosis. Curr Atheroscler Rep..

[23] Ortega-Gutierrez S, Holcombe A, Aksan N, Dai B, Shaban A, Lazarre L (2020). Association of admission clinical predictors and functional outcome in patients with Cerebral Venous and Dural Sinus Thrombosis. Clin Neurol Neurosurg..

[24] Simaan N, Molad J, Peretz S, Filioglo A, Auriel E, Hallevi H (2022). Characteristics of Cerebral Sinus Venous Thrombosis Patients Presenting with Intracerebral Hemorrhage. J Clin Med..

[25] Silvis SM, de Sousa DA, Ferro JM, Coutinho JM (2016). Cerebral venous thrombosis. Presse Med..

[26] Agrawal K, Burger K, JF R. (2016). Cerebral Sinus Thrombosis. Headache.

[27] Aguiar de Sousa D, Pereira-Santos MC, Serra-Caetano A, Lucas Neto L, Sousa AL, Gabriel D (2021). Blood biomarkers associated with inflammation predict poor prognosis in cerebral venous thrombosis: a multicenter prospective observational study. Eur J Neurol..

[28] Zhang X, Ding R, Li H, Liu Y, Ou W, Hu J (2021). An Association between Inflammation and Cerebral Venous Thrombosis: A Retrospective Study. J Stroke Cerebrovasc Dis..

[29] Kolaczkowska E, Kubes P (2013). Neutrophil recruitment and function in health and inflammation. Nat Rev Immunol..

[30] Tekesin A, Tunc A (2019). Inflammatory markers are beneficial in the early stages of cerebral venous thrombosis. Arq Neuropsiquiatr..

[31] Kamisli O, Kamisli S, Kablan Y, Gonullu S, Ozcan C (2013). The prognostic value of an increased mean platelet volume and platelet distribution width in the early phase of cerebral venous sinus thrombosis. Clin Appl Thromb Hemost.

[32] Maino A, Abbattista M, Bucciarelli P, Artoni A, Passamonti SM, Lanfranconi S (2017). Red cell distribution width and the risk of cerebral vein thrombosis: A case–control study. European Journal of Internal Medicine.

[33] Artoni A, Abbattista M, Bucciarelli P, Gianniello F, Scalambrino E, Pappalardo E (2018). Platelet to Lymphocyte Ratio and Neutrophil to Lymphocyte Ratio as Risk Factors for Venous Thrombosis. Clin Appl Thromb Hemost..

[34] Akboga YE, Bektas H, Anlar O (2017). Usefulness of platelet to lymphocyte and neutrophil to lymphocyte ratios in predicting the presence of cerebral venous sinus thrombosis and in-hospital major adverse cerebral events. J Neurol Sci..

[35] Lehmann F, Schenk LM, Bernstock JD, Bode C, Borger V, Gessler FA (2021). Elevated Red Cell Distribution Width to Platelet Ratio Is Associated With Poor Prognosis in Patients With Spontaneous, Deep-Seated Intracerebral Hemorrhage. Front Neurol..

[36] Kline JA, Zeitouni R, Hernandez-Nino J, GA R. (2008). Size isn't everything. J Thromb Haemost..

[37] Kim YD, Song D, Nam HS, Lee K, Yoo J, Hong GR (2015). D-dimer for prediction of long-term outcome in cryptogenic stroke patients with patent foramen ovale. Thromb Haemost..

[38] Dentali F, Squizzato A, Marchesi C, Bonzini M, Ferro JM, Ageno W (2012). D-dimer testing in the diagnosis of cerebral vein thrombosis: a systematic review and a meta-analysis of the literature. J Thromb Haemost..

[39] Heldner MR, Zuurbier SM, Li B, Von Martial R, Meijers JCM, Zimmermann R (2020). Prediction of cerebral venous thrombosis with a new clinical score and D-dimer levels. Neurology..

