# Supplementary material for: Association of neutrophil to lymphocyte ratio and D-dimer with functional outcome in patients with cerebral venous sinus thrombosis
Source: BMC Neurol. 2023 Jan 19;23:30. doi: 10.1186/s12883-022-03030-4 (PMC9850605; doi:10.1186/s12883-022-03030-4)
Supplement: Supplementary file 1 — Additional file 1: Supplementary Table 1. Collinearity test for clinical indexes [file 12883_2022_3030_MOESM1_ESM.doc]

Supplementary Table 1. Collinearity test for clinical indexes

| **Variable** | **Tolerance** | **VIF** |  |
| --- | --- | --- | --- |
|  |
| Impaired consciousness | 0.941 | 1.063 |  |
| Brain lesion | 0.976 | 1.024 |  |
| D-dimer | 0.676 | 1.480 |  |
| RDW | 0.859 | 1.164 |  |
| Neutrophil | 0.492 | 2.031 |  |
| Lymphocyte | 0.641 | 1.560 |  |
| NLR | 0.396 | 2.525 |  |
| RPR | 0.660 | 1.516 |  |
| RDW, red cell distribution width; NLR, neutrophil to lymphocyte ratio; RPR, red blood cell distribution width to platelet ratio; VIF, variance inflation factor. | | |  |
|  | | |  |
|  | | |  |
